# The Year in Cardiology 2015: Imaging

**DOI:** 10.1093/eurheartj/ehv732

**Published:** 2016-10

**Authors:** Oliver Gaemperli, Victoria Delgado, Gilbert Habib, Philipp A. Kaufmann, Jeroen J. Bax

**Affiliations:** 1 Cardiac Imaging - University Heart Center, Zurique, Suíça; 2 Heart Lung Centrum - Leiden University Medical Center, Leiden, Países Baixos; 3 Service de Cardiologie - C.H.U. De La Timone - Bd Jean Moulin, Marselha, França; 4 Department of Nuclear Medicine, Cardiac Imaging, University Hospital Zurich, Zurique, Suíça

**Keywords:** Cardiovascular Diseases / diagnosis, Diagnostic Imaging / trends, Magnetic Resonance Spectroscopy / methods, Echocardiography / methods

## Preamble

Prognostic implications of several non-invasive imaging techniques have been the focus of some landmark studies published in 2015. Non-invasive characterization of atherosclerosis processes and vulnerable plaques have been possible with advances in cardiac magnetic resonance imaging and nuclear imaging techniques. In addition, 3-dimensional echocardiography and multidetector-row computed tomography have improved our understanding of valvular heart disease. Finally, data on the clinical role of integration of non-invasive imaging techniques (fusion imaging) are accumulating and its use is expected to increase in the coming years. The current review provides a summary of selected articles on prognostic impact of current non-invasive imaging techniques and technological innovations.

### Echocardiography

In 2015, the new recommendations for cardiac chamber quantification using echocardiography in adults were published providing updated normative values for all four cardiac chambers based on multiple databases compiling data from a large number of normal subjects.^1^ In addition, this position document includes reference values for chamber quantification with three-dimensional (3D) echocardiography and myocardial deformation with strain imaging. These normative data permit differentiation between normal and abnormal findings. From a clinical perspective however, definition of the degree of abnormality (mild, moderate, or severe) may be more meaningful. The document acknowledges the difficulties to determine cut-off values that define the degree of abnormality and provides experience-based partition values only for left ventricular (LV) size, function and mass, and for left atrial (LA) volume.

Data showing the prognostic value of LV global longitudinal strain (GLS) are accumulating. A recent meta-analysis pooling data from 16 studies (n = 5721), encompassing different cardiac diseases [heart failure, acute myocardial infarction (MI), and valvular heart disease among others], showed that the prognostic value of LV GLS exceeds that of LV ejection fraction (EF).^2^ On multivariable analysis, each 1 standard deviation (SD) change in LV GS was independently associated with all-cause mortality (hazard ratio, HR 0.50; 95% CI 0.36-0.69) compared with LVEF (HR 0.81; 95% CI 0.72-0.92), indicating that the HR per each 1 SD change in LV GLS was 1.62 times greater than that of LVEF (95% CI 1.13-2.33; p = 0.009). In patients with MI, regional LV longitudinal strain may clinically be more meaningful than GLS. A subanalysis of the VALIANT (Valsartan in Acute Myocardial Infarction Trial) trial including 248 patients with LV systolic dysfunction, heart failure, or both demonstrated that regional LV longitudinal strain was significantly impaired even in segments with normal wall motion score index compared with healthy controls (−10.4 ± 5.2% vs. −20.0 ± 7.6, p < 0.001).^3^ Abnormal longitudinal strain segments were defined as having a strain value higher (less negative) than the 95% percentile of corresponding normal control segments. An increasing number of LV segments with abnormal regional strain was associated with an increased risk of all-cause mortality (HR 1.42; 95% CI 1.06-1.90, p < 0.001).

Echocardiographic assessment of heart failure patients who are candidates for cardiac resynchronization therapy (CRT) remains of interest. Results of the EchoCRT (CRT in heart failure with narrow QRS complex) substudy showed that 77% of the 614 patients with echocardiographic follow-up at 6 months had persistent or worsened LV dyssynchrony (≥130 ms as measured with STE or ≥80 ms using tissue Doppler imaging).^4^ The presence of persistent or worsened LV dyssynchrony was associated with increased risk of all-cause mortality and heart failure hospitalization (HR 1.54, 95% CI 1.03-2.3; p = 0.02). These results were also observed in the large multicentre registry PREDICT-CRT.^5^ Left ventricular apical rocking and septal flash visualized on echocardiography are markers of left bundle branch block-induced LV dyssynchrony. In 1060 patients treated with CRT, correction of apical rocking and septal flash at 6-12 months echocardiography was associated with LV reverse remodelling and better survival at long-term follow-up. In contrast, patients who still exhibited LV apical rocking or septal flash at follow-up showed less LV reverse remodelling and worse outcome.

The prognostic value of right ventricular (RV) function was also evaluated in several studies.^6,7^ In 96 patients with heart failure with preserved LVEF and 46 controls who underwent clinically indicated right-sided heart catheterization and transthoracic echocardiography, Melenovsky and coworkers showed that male gender, LVEF, atrial fibrillation, coronary artery disease (CAD), and systemic systolic blood pressure were independently associated with RV dysfunction (defined as RV fractional area change <35%) after adjusting for RV pulmonary arterial pressures.^6^ Patients with RV dysfunction showed lower 2-year survival compared with patients without (56 vs. 93%). Right ventricular dysfunction was the strongest associate of all-cause mortality in a model corrected for systolic pulmonary artery pressure (HR 2.2; 95% CI 1.4-3.5; p = 0.001). Advances in 3D strain imaging have allowed characterization of RV remodelling in patients with pulmonary hypertension.^7^ Right ventricular morphological and functional data of 92 patients with pulmonary hypertension were analysed with novel 3D motion tracking echocardiography. Based on pressure-volume loops obtained during right-sided heart catheterization, patients were divided into three groups: RV adapted, RV adapated-remodelled, and RV adverse-remodelled. A progressive impairment in RVEF and global area strain was observed across the three groups with the RV adverse-remodelled group having the worst values. Patients within the RV adapted group showed better 6-month free-survival from hospitalization, death, or lung transplantation compared with the other groups (HR 0.15; 95% CI 0.07-0.3; p < 0.001), whereas patients within the RV adverse-remodelled group showed the worst outcome (HR 2.2; 95% CI 0.91-5.39, p = 0.004).

**Figure 1:**
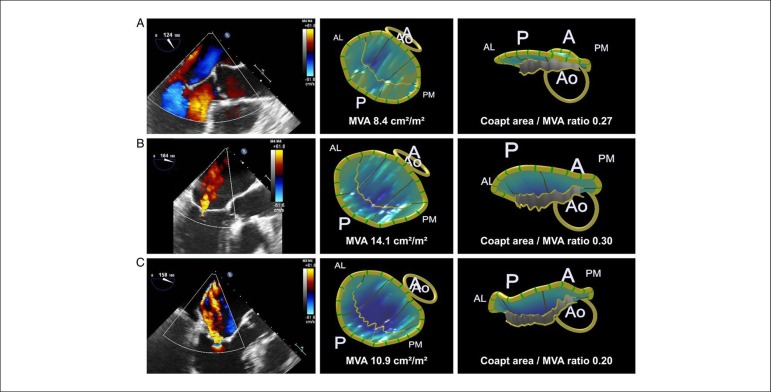
Examples of mitral valve leaflet remodelling using 3D transoesophageal echocardiography. (A) Example of an individual without functional mitral regurgitation. (B) Examples of two patients with mild (B) and severe functional mitral regurgitation (C), both secondary to inferior infarction. Note the larger mitral valve area (MVA) as well as the coaptation area to MVA ratio in the patient with mild vs. the patient with severe functional mitral regurgitation. A: anterior; AL: anterolateral; Ao: aorta; P: posterior; PM: posteromedial. Reproduced with permission from Debonnaire et al.8 This Figure has been reprinted by permission of Oxford University Press on behalf of the European Society of Cardiology.

Three-dimensional echocardiography is increasingly used in heart valve disease. Debonnaire and coworkers demonstrated that 3D transoesophageal echocardiography could adequately depict mitral valve leaflet remodelling in patients with LV dysfunction and functional mitral regurgitation (MR).^8^ Insufficient leaflet remodelling relative to annular and LV dilatation resulted in reduced coaptation, which was independently associated with MR severity in patients with functional MR. Figure 1 illustrates different examples of mitral valve leaflet remodelling in patients with functional MR and patients without MR. Clavel et al. used 3D transoesophageal echocardiography to evaluate 49 patients with degenerative mitral valve disease.^9^ The authors demonstrated important differences in LV remodelling, annular, and valvular dimensions, associated with differences in MR severity between patients with fibro-elastic deficiency and diffuse myxomatous degeneration.

Finally, the incremental value of quantification of tricuspid regurgitation (TR) to predict survival was demonstrated by Topilsky et al.^10^ In 353 patients with various degrees of isolated functional TR, the effective regurgitant orifice area (EROA) was calculated with the proximal flow convergence method. An EROA ≥40 mm^2^ defined severe TR and was observed in 40% of patients. During a mean follow-up of 5.8 years, 82 patients died. An EROA ≥40 mm^2^ was independently associated with all-cause mortality (HR 2.95; 95% CI 1.67-5.19; p < 0.001) after adjusting for clinical characteristics, LVEF, RV size, RV function, and RV systolic pressure.

### Computed tomography

Very long-term prognostic data for coronary artery calcium score (CACS) were published by Valenti and coworkers in 9715 asymptomatic subjects followed for 14.6 years: CACS emerged as the strongest predictor of death, and was independent from Framingham risk score (FRS) or National Cholesterol Education Program Adult Treatment Panel III risk category.^11^ A CACS = 0 was associated with an invariably low death rate of 4.7% (i.e. 0.3%/year on average), and thereby extends the warranty period of zero CACS over a period of almost 15 years, particularly in low and intermediate risk patients, and regardless of gender. In a recent publication from the COronary CT Angiography EvaluatioN For Clinical Outcomes: An InteRnational Multicenter registry, investigators observed in 3217 asymptomatic subjects that CT coronary angiography (CTCA) possessed incremental prognostic value over FRS only in patients with a CACS >100 (net reclassification improvement 0.62, p < 0.001), but not among those with CACS ≤100.^12^ However, in high and very high CACS subgroups (i.e. >400 and >1000), the incremental value of CTCA was lost again, probably through less reliable CTCA interpretation. However, conclusions in these subgroups were limited due to low sample sizes and event rates.

The Coronary computed tomography angiography (CTA) vascular events in non-cardiac surgery patients cohort evaluation (VISION) study assessed the value of CTCA for predicting the risk of cardiovascular complications of non-cardiac surgery.^13^ A total of 955 patients with vascular risk factors were included, of which 74 (8%) suffered a perioperative event (cardiovascular death/MI). Computed tomography coronary angiography findings provided independent prognostic information over revised cardiac risk indices with increasing HRs for non-obstructive (HR 1.51, p = 0.30), obstructive (HR 2.05, p = 0.076), and extensive obstructive (HR 3.76, p < 0.001) disease. However, the c-index increased only from 0.62 to 0.66 when adding CTCA to the clinical risk prediction models, mostly because of reclassification of a sizable number of patients (~10%) into a higher-risk category who would not suffer any subsequent event. Thus, the results of the coronary CTA VISION raise concerns about overestimation of risk by CTCA compared with clinical risk indices.

The diagnostic yield of CT for triple rule-out (TRO) of MI, pulmonary embolism (PE), and aortic dissection (AD) in patients with acute chest pain is a debated issue, and was investigated in the Advanced Cardiovascular Imaging Consortium database in 12 834 patients.^14^ The overall diagnostic yield was similar for TRO CT compared with CTCA only (17.4 vs. 18.3%; P = 0.37) and was driven mainly by CAD detection (15.5 vs. 17.2%, p = 0.093); TRO CT, however, yielded slightly more PE (1.1 vs. 0.4%; p = 0.004) and AD (1.7 vs. 1.1%; p = 0.046) diagnoses, although at a higher median radiation (9.1 vs. 6.2 mSv; p < 0.0001) and mean contrast dose (113 ± 6 vs. 89 ± 17 mL; p < 0.0001), and higher non-diagnostic image quality rate (9.4 vs. 6.5%; p < 0.0001). Thus, although TRO CT may be useful in selected patients (after careful consideration of individual MI/PE/AD risks), the study does not support its indiscriminate use in emergency departments.

The results of two large randomized CT studies were eagerly awaited in 2015: in the prospective multicenter imaging study for evaluation of chest pain trial, 10 003 symptomatic patients with intermediate CAD pretest probability were randomized to a strategy of initial anatomical testing with CTCA vs. traditional functional testing (67% stress nuclear, 23% stress echocardiography, and 10% exercise ECG).^15^ At 25 months follow-up, there were no differences in the primary endpoint of death, MI, hospitalization for unstable angina, or major procedural complications. However, CTCA resulted in fewer catheterizations showing no obstructive CAD (3.4 vs. 4.3%, p = 0.02), although more patients in the CTCA group underwent catheterization (12.2 vs. 8.1%) and were revascularized (6.2 vs. 3.2%, p < 0.001) within 90 days of randomization. The Scottish COmputed Tomography of the HEART trial randomized 4146 patients to standard care (SC) (including clinical assessment and exercise ECG) plus CACS and CTCA vs. SC alone.^16^ The use of CTCA increased diagnostic certainty [relative risk (RR) 1.79; p < 0.001) for the primary endpoint of angina due to CAD, resulted in cancellation of 121 functional tests and 29 invasive angiograms, and more changes in preventive and antianginal drug therapies. At follow-up of 1.7 years, there was a numerical (although not significant) 38% reduction of the composite endpoint of CAD death/MI in the CTCA group (p = 0.053).

**Figure 2:**
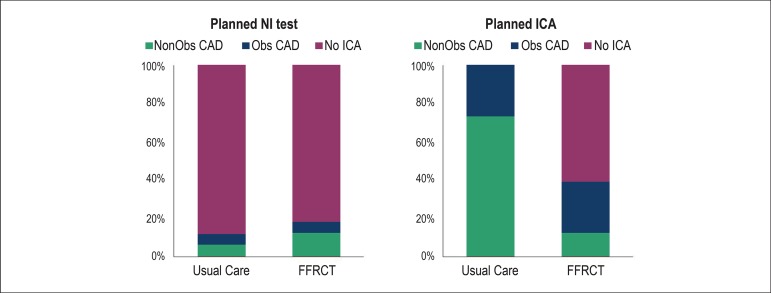
The PLATFORM (Prospective LongitudinAl Trial of FFRct: Outcome and Resource IMpacts) study compared FFRCT as gatekeeper for invasive coronary angiography with direct angiography (right panel), as well as FFRCT vs. routine non-invasive testing as gatekeeper for invasive angiography (left panel). In the patients with planned invasive coronary angiography (right panel), the use of FFRCT as gatekeeper avoided invasive coronary angiography in 61%, and reduced the percentage of non-obstructive coronary artery lesions from 73 to 12%, whereas there were no differences in percentage of non-obstructive lesions on invasive angiography in the patients undergoing planned non-invasive testing (left panel). NI: non-invasive; ICA: invasive coronary angiography; Obs CAD: obstructive coronary artery disease; FFRCT: computation of fractional flow reserve from coronary computed tomographic angiography data. Reprinted from Douglas et al.17 This Figure has been reprinted by permission of Oxford University Press on behalf of the European Society of Cardiology.

Computed tomography-derived fractional flow reserve (FFR_CT_) continues to raise interest in 2015 through its latest publication, the Prospective LongitudinAl Trial of FFRct: Outcome and Resource IMpacts study.^17^ In this trial, 584 symptomatic patients with intermediate CAD pretest probability were prospectively (but not randomly) assigned to receive either usual testing (n = 287, i.e. non-invasive testing or invasive coronary angiography, ICA) or CTCA (n = 297) with additional FFR_CT_ where requested. Among those with intended ICA (n = 380), FFR_CT_ resulted in a significant reduction in the number of invasive catheterizations showing no obstructive CAD (from 73 to 12%) and avoided ICA in 117 (61%) patients, while no differences were noted in the group of patients with intended non-invasive testing (Figure 2). Although the PLATFORM study was not randomized, it provides a contemporary snapshot of the current use of diagnostic 'platforms' for CAD work-up, and suggests overuse of ICA in intermediate probability patients which could be reduced by wider use of FFR_CT_. Interestingly, the recently published PLATFORM substudy demonstrated that the use of FFR_CT_ was associated with $3391 costs reduction compared with ICA, whereas differences in downstream costs between FFR_CT_strategy and usual care (non-invasive testing) were not significant ($7047 vs. $8422, respectively).^18^ However, in the non-invasive arm, patients undergoing FFR_CT_ showed better scores on quality-of-life questionnaires compared with patients undergoing usual care, whereas in the invasive arm, there were no differences between FFR_CT_ and ICA.

### Cardiac magnetic resonance

**Figure 3:**
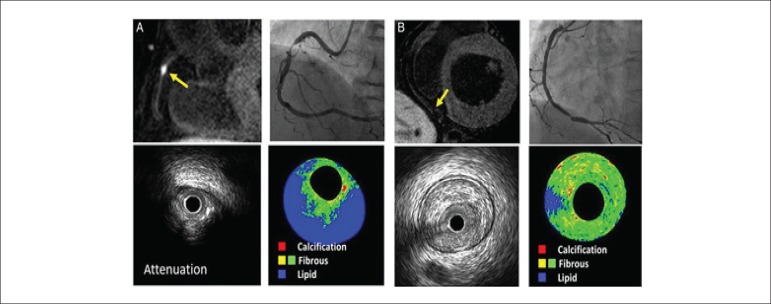
Coronary artery plaque characteristics assessed with non-contrast T1-weighted cardiac magnetic resonance (CMR) imaging. (A) A significant stenosis of the mid-right coronary artery (on invasive angiography). On non-contrast T1-weighted CMR (upper left corner), a high-intensity plaque can be observed (plaque to myocardium intensity ratio of 3.09) which shows attenuation and lipid-rich composition on intravascular ultrasound. (B) A significant stenosis of the distal right coronary artery and non-high-intensity plaque on CMR. Intravascular ultrasound with virtual histology shows a fibrous plaque. Reproduced with permission from Hoshi et al.19 This Figure has been reprinted by permission of Oxford University Press on behalf of the European Society of Cardiology.

Characterization of coronary artery plaques with non-contrast T1-weighted magnetic resonance imaging (MRI) has provided novel insights into the pathophysiology of percutaneous coronary intervention (PCI)-related myocardial injury, a procedural complication which has important prognostic implications.^19^ Seventy-seven patients with stable angina and significant coronary artery lesions (>70% stenosis on invasive angiography) underwent 1.5T MRI 48 h prior to PCI and coronary plaque composition was assessed with non-contrast T1-weighted MRI. High-intensity plaques (considered vulnerable plaques) were defined by a 'coronary plaque to myocardium signal intensity' ratio of ≥1.4. Percutaneous coronary intervention-related myocardial injury was defined as an increased high-sensitivity cardiac troponin T >5 times the 99th percentile upper reference limit. Patients with high-intensity plaques (n = 31) showed greater plaque burden, larger lipid pool, more frequently positive remodelling, ultrasound attenuation, and intracoronary thrombus on intravascular ultrasound analysis, compared with patients without high-intensity plaques (Figure 3). Importantly, the presence of high-intensity plaques was associated with higher frequency of PCI-related myocardial injury (58 vs. 11%, p < 0.001). How this information may influence the decision making and interventional strategy needs further study.^20^

In survivors of ST-segment elevation acute MI (STEMI), assessment of infarct size and microvascular obstruction with contrast-enhanced CMR has important prognostic value. Interestingly, non-contrast CMR-derived parameters such as native T1 mapping permit characterization of the infarct core tissue. After MI, there is an increase in water content in the ischaemic area that will result in longer native T1 times. Carrick and coworkers investigated in 300 survivors of STEMI, the correlation between native T1 time of the infarct core, infarct size and microvascular obstruction and the prognostic implications of native T1 time in terms of LV adverse remodelling (≥ 20% increase in end-diastolic volume at 6 months follow-up), all-cause mortality and heart failure hospitalization.^21^ Patients underwent cine CMR, native T1 mapping, T2 mapping, T2* mapping and late gadolinium contrast-enhanced (LGE) sequences 2 days after index MI and at 6 months follow-up. Native T1 times were measured in the infarct zone, injured myocardium, and remote myocardium. The infarct zone region was defined as myocardium with pixel values (T1 or T2) >2 SD from the remote zone on T2-weighted CMR sequences. The hypo-intense infarct core was defined as areas within the infarct zone with pixel T1 values <2 SD of the values observed in periphery of the infarct zone. Infarct core native T1 was significantly associated with native T2 (r = 0.42, p < 0.001). Native T1 values within the infarct core were associated with LV adverse remodelling at 6 months follow-up (odds ratio 0.91 per each 10 ms reduction, p = 0.061) and were independent predictors of all-cause mortality or heart failure hospitalization (HR 0.73, p < 0.001).

Several CMR-derived variables have been used to define and quantify the presence of myocardial fibrosis (focal or diffuse). While focal macroscopic fibrosis is commonly assessed with LGE CMR, diffuse fibrosis can be characterized by calculating the extracellular volume (ECV), after primed contrast infusion or administration of bolus of gadolinium, post-contrast T1-mapping values and pre-contrast (native) T1-mapping values. In addition, changes in T1-weighted LGE-signal intensity have been associated with myocardial injury. These measures have been correlated with LV function and prognosis in several cardiac diseases.^22-26^ In 65 patients who underwent potentially cardiotoxic chemotherapy, LVEF decreased significantly after 3 months of therapy (from 57 ± 1 to 54 ± 1%, p < 0.001) while myocardial T1-weighted LGE-signal intensity increased (from 14.1 ± 0.6to 15.9 ± 0.8, p = 0.046) without an increase in myocardial oedema on T2-weighted CMR sequences.^22^ Whether these changes may predict irreversible myocardial damage after withdrawal of chemotherapy, remains unknown. The presence of subclinical diffuse myocardial fibrosis was evaluated measuring the ECV in 35 patients with asymptomatic moderate and severe primary MR and preserved LVEF.^23^ Compared with controls, patients with MR exhibited larger ECV (0.32 ± 0.07 vs. 0.25 ± 0.02, p < 0.01). Increasing myocardial ECV was significantly associated with larger LV end-systolic volume index (r = 0.62, p < 0.001) and left atrial volume index (r = 0.41, p < 0.05), lower LVEF (r = −0.6, p < 0.001) and worse functional capacity as measured with peak oxygen consumption (r = −0.51, p < 0.005). In 139 patients with hypertrophic cardiomyopathy (HCM), Ellims et al. investigated the correlations of macroscopic myocardial fibrosis (assessed with LGE CMR) and diffuse myocardial fibrosis (assessed with post-contrast T1 mapping) with LV function and genotype.^24^ The presence of LGE was associated with LVEF and presence of LV outflow tract obstruction whereas shorter post-contrast T1-mapping values (more diffuse fibrosis) were significantly associated with LV diastolic dysfunction and dyspnoea symptoms. Interestingly, patients with identifiable HCM genetic mutations showed larger extent of LGE (7.9 ± 8.6 vs. 3.1 ± 4.3%, p = 0.03) but longer post-contrast T1-mapping values (498 ± 81 vs. 451 ± 70 ms, p = 0.03) compared with patients without mutations. Using LGE CMR, Nadel et al. investigated in 106 patients with biopsy-proven extracardiac or cardiac sarcoidosis the association between the presence of focal macroscopic fibrosis and occurrence of the composite endpoint all-cause mortality, sudden cardiac death, ventricular tachycarrhyhtmia or ventricular fibrillation.^25^ Thirty-two (30%) patients showed focal myocardial fibrosis on LGE CMR which was of patchy distribution in the majority of patients (72%) followed by subepicardial (59%) and midwall (25%) distribution. During a mean follow-up of 37 months, 16 patients reached this composite endpoint. The presence of focal myocardial fibrosis on LGE CMR was independently associated with the composite endpoint (HR 12.52, 95% CI 1.35-116.18, p = 0.03). In 100 patients with systemic light-chain amyloidosis, Banypersad and coworkers evaluated the prognostic value of ECV, pre-contrast (native), and post-contrast T1 values.^26^ During a median follow-up of 23 months, 25% of patients died. A cut-off value of ECV ≥0.45 (HR 3.84, 95% CI 1.53-9.61, p = 0.004) and a cut-off value of native T1 time ≥1044 ms (HR 5.39, 95% CI 1.24-23.4, p = 0.02) were independently associated with all-cause mortality, whereas post-contrast T1 mapping values were not predictive of mortality.

### Nuclear imaging

In patients with cardiovascular risk factors, characterization of inflammation and lipid accumulation in the arterial wall using nuclear imaging has been the target of several studies.^27,28^ Van der Falk et al. aimed at assessing the role of leukocytes in atherogenesis by performing single photon emission computed tomography (SPECT)-CT with ^99m^Technetium-labeled peripheral blood mononuclear cells (PBMC).^27^ In 10 patients with known cardiovascular disease and 5 healthy controls, a markedly enhanced accumulation of PBMC was found in patients with advanced atherosclerotic lesions. This represents a novel-imaging approach to visualize leukocyte migration and PBMC accumulation to atherosclerosis in humans, potentially lending support to strategies aimed at attenuating leukocyte recruitment as a therapeutic target in patients with cardiovascular disease. Van Wik et al. demonstrated that lipoprotein apheresis leads to a marked reduction of arterial wall inflammation in patients with familial hypercholesterolaemia (FH) characterized by severely elevated plasma low-density lipoprotein cholesterol levels.^28^ 18-F-fluorodeoxyglucose (FDG)-positron emission tomography (PET) was used to assess the target-to-background ratio (TBR) of FDG uptake within the arterial wall in 24 patients with known FH and in 14 normolipidemic controls. A second PET scan was acquired after 3 days in 12 patients in whom lipoprotein apheresis was performed and demonstrated a significant reduction of TBR compared with the baseline scan (2.05 ± 0.31 vs. 1.91 ± 0.33; p < 0.02). These suggest that apoprotein B-containing lipoproteins play a role in arterial wall inflammation and support the concept of a beneficial effect of lipoprotein apheresis.

In addition, Moon et al. sought to investigate the added prognostic value of FDG-PET over the FRS and carotid intima-media thickness (CIMT) for the prediction of future cardio-cerebrovascular events.^29^ Carotid FDG uptake and CIMT were measured in 1089 asymptomatic adults who underwent PET imaging. Cardio-cerebrovascular events occurred in 19 participants (1.74%) during an average follow-up of 4.2 years. Multivariate Cox regression analysis revealed that high carotid FDG uptake (HR 2.98; 95% CI 1.17-7.62; p = 0.022) and high CIMT (HR 2.82; 95% CI 1.13-7.03; p = 0.026) were independent predictors of these events. Furthermore, carotid FDG uptake improved discrimination of risk prediction when added to the FRS independently CIMT.

In patients with suspected acute coronary syndrome and negative cardiac biomarkers routine exercise testing is recommended by current guidelines.^30^ Little is known on the potential role of nuclear myocardial perfusion imaging (MPI) in this context. Cremer et al. reported on the yield of SPECT-MPI for detecting ischaemia, its prognostic value for short-term events, and its impact on downstream resource utilization.^31^ Among 5354 patients referred from the emergency department after negative troponin T tests and non-diagnostic ECGs, only 6.1% of patients with a thrombolysis in myocardial infarction (TIMI) score ≤ 2 presented with > 5% ischaemic myocardium, while 19.6% of patients with TIMI scores ≥ 3 had >5%. Furthermore, short-term adverse events were rare at 30 days with only 0.1% mortality and 0.1% of patients undergoing revascularization for acute MI. These findings suggest that SPECT-MPI before discharge after two negative troponins should be helpful in patients with TIMI scores ≥ 3.

In the field of acute MI, it has been shown that FDG-PET may be able to detect inflammation in the acutely infarcted myocardium, if information on late contrast enhancement (scar tissue) from concomitant CMR or CT is integrated. Wollenweber et al. have translated these concepts into 15 patients early after MI by performing PET and CMR within 7 days of first MI.^32^ All patients underwent heparin pre-treatment to suppress FDG uptake in remote myocardium. The metabolic rate of glucose was significantly increased in infarcted vs. remote myocardium (2.0 vs. 0.4 mg/min per 100 mL; p = 0.0001). Regionally, FDG score was highest in segments with LGE vs. oedema only and to remote myocardium (2.0 vs. 1.8 vs. 0.4; p < 0.0001). Thus, increased FDG uptake after heparin-induced suppression of myocyte uptake appears to reflect inflammatory activity in acutely infarcted myocardial tissue.

Finally, the radiation burden associated with nuclear imaging remains of concern and was the objective of the INCAPS Investigators Group which conducted an observational cross-sectional study of nuclear MPI protocols in 308 nuclear cardiology laboratories in 65 countries around the world, characterizing patient radiation doses and the use of radiation optimizing 'best practices'.^33^ Patient effective radiation dose ranged between 0.8 and 35.6 mSv (median 10.0 mSv). Average laboratory effective dose ranged from 2.2 to 24.4 mSv (median 10.4 mSv) and only 30% of all laboratories achieved a median effective dose of ≤ 9 mSv as recommended by guidelines. The lowest effective dose (median 8.0 mSv) was administered in Europe, coinciding with the highest best-practice adherence rate.

### Integration or fusion of different imaging modalities

The number of studies published on the use of integrated or fusion imaging is increasing, indicating increasing use of integrated PET-CT and PET-MRI equipment, but also the fusion of independently obtained data from (for example) SPECT and fluoroscopy. Zhou and colleagues developed a 3D fusion tool kit to fuse LV venous anatomy (derived from fluoroscopy) with SPECT-MPI (to assess myocardial scar) to guide LV lead placement in CRT.^34^

Fusion imaging with PET-CT has been applied in patients with suspected CAD.^35,36^ Valenta and colleagues evaluated 24 patients with PET-CT using N13-ammonia to assess different myocardial blood flow variables (flow reserve and flow gradients), which were related with CTCA, resulting in better understanding of the haemodynamic significance of coronary stenoses.^35^ Dey et al. reported on PET-CT data from 51 patients: CTCA (quantitatively analysed) was fused with myocardial flow reserve (derived from rest-stress N13-ammonia PET).^36^ Prediction of reduced myocardial flow reserve (indicating ischaemia) was optimal when CTCA stenosis severity was integrated with various other CTCA variables, including total (non-calcified) plaque burden. These findings indicate that an integrated CTCA score (including stenosis severity, total plaque burden, and plaque constitution) may better predict reduced myocardial flow reserve.

**Figure 4:**
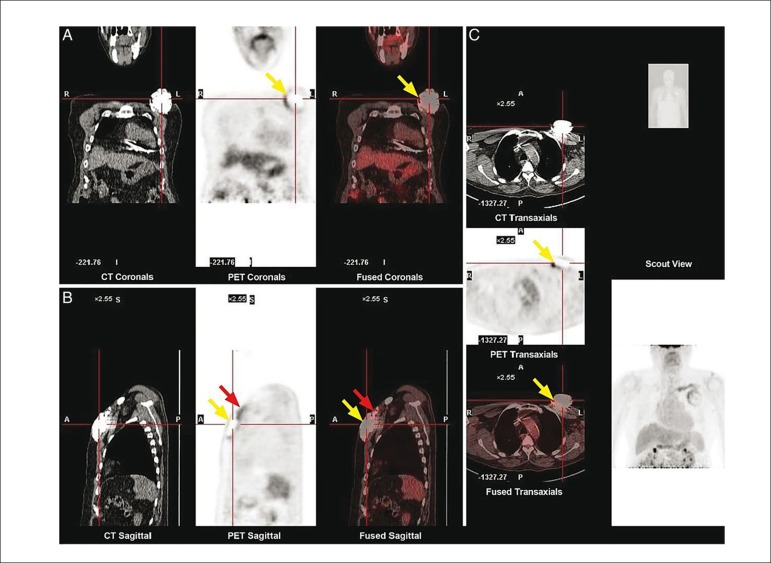
PET-CT in suspected device pocket infection. Example of a positive 18F-FDG PET/CT scan in a patient with pain at the generator pocket site. (A) Increased FDG uptake is seen in the region of the left pre-pectoral pocket on the coronal views (yellow arrows). (B) In the sagittal plane, increased FDG uptake can be seen on the muscular aspect of the pre-pectoral generator (yellow arrows) and along the proximal portion of the leads (red arrows). (C) Increased FDG uptake visualized on the muscular aspect of the generator pocket (yellow arrows). Reproduced with permission from Ahmed et al.37 This Figure has been reprinted by permission of Oxford University Press on behalf of the European Society of Cardiology.

Ahmed et al. explored the utility of ^18^F-FDG-PET-CT in the diagnosis of cardiac implantable electronic device generator pocket infection.^37^ To this end, 46 patients with suspected generator pocket infection and 40 without any infection underwent PET imaging, and FDG activity in the region of the generator pocket (Figure 4) was expressed as a semi-quantitative ratio (SQR) defined as the maximum count rate around the generator divided by the count rate between normal right and left lung parenchyma. Patients with suspected generator pocket infection that required generator extraction had significantly higher FDG activity compared with those that did not, and with controls (SQR 4.80 vs. 1.40 vs. 1.10, p < 0.001). From receiver operator characteristic curve analysis, the authors calculated an optimal SQR cut-off value of >2.0, yielding a very high sensitivity and specificity of 97 and 98%, respectively. These results demonstrate a high diagnostic performance and highlight the potential utility of FDG-PET for the detection of early cardiac implantable electronic device generator pocket infection.

**Figure 5:**
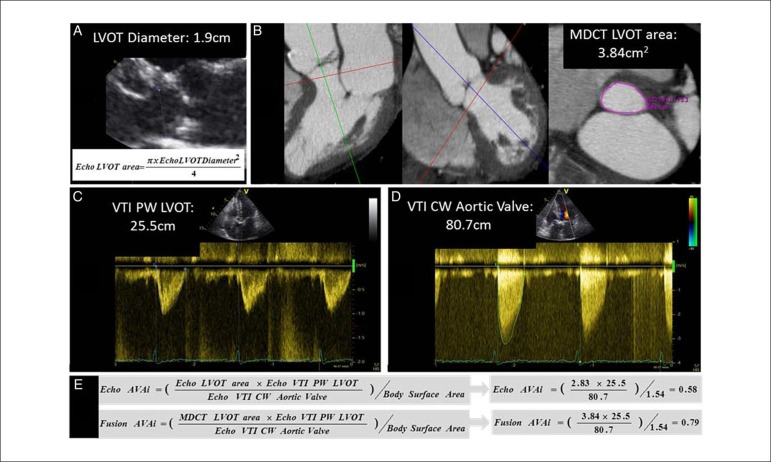
Quantification of aortic valve area using fusion imaging in aortic stenosis. Current clinical practice, 2-dimensional and Doppler echocardiography are used to calculate the aortic valve area (panels A, C, D and E): the LV outflow tract (LVOT) diameter is measured from the parasternal long-axis view and the flow of the LVOT and gradient of aortic valve are measured with pulsed and continuous wave Doppler. By introducing the true cross-sectional area of the LVOT measured with MDCT (panel B) into the Bernoulli equation (panel E), the aortic valve area fusion is calculated. In this particular example, an aortic valve area index of 0.58 cm^2^/m^2^ calculated with echocardiography (Echo AVAi) indicates severe aortic stenosis whereas by using the MDCT cross-sectional area of the LVOT, the aortic valve area index (Fusion AVAi) increases to 0.79 cm2/m2 indicating moderate aortic stenosis. Reproduced with permission from Kamperidis et al.38 This Figure has been reprinted by permission of Oxford University Press on behalf of the European Society of Cardiology.

Fusion imaging of CT and echocardiography in heart valve disease was reported by Kamperidis et al.^38^ The authors addressed the topic of low gradient, but severe aortic stenosis in patients with preserved LVEF; this 'mismatch' between the low gradient over the valve (indicating no stenosis) but the small valve area (indicating severe stenosis) may be related to the assumption of a circular shape of the LV outflow tract with 2D echocardiography (which in fact often may have an elliptical shape). Since this parameter contributes significantly to the calculation of the aortic valve area (Figure 5), this may contribute to errors in classification of severity of aortic stenosis. The LV outflow tract may be more accurately detected from CT (anatomical) imaging by direct planimetry, and fusion of the CT-derived LV outflow tract area with the echo Doppler data may result in significant reclassification of inconsistently graded severe aortic stenosis. In 191 patients with severe aortic valve stenosis (according to the aortic valve area indexed for body surface area being < 0.6 cm^2^/m^2^) and preserved LVEF (≥ 50%), this fusion approach was applied and reclassified 52% of patients with low gradient but severe aortic stenosis and preserved LVEF into moderate aortic stenosis (Figure *5*).
